# Identification of patients with moderate medically unexplained physical symptoms in primary care with a five years follow-up

**DOI:** 10.1186/s12875-019-0950-7

**Published:** 2019-05-21

**Authors:** Paula Elisabeth van Westrienen, Martijn Frits Pisters, Cindy Veenhof, Nicolaas Johannes de Wit

**Affiliations:** 1 Center for Physical Therapy Research and Innovation in Primary Care, Leidsche Rijn Julius Health Care Centers, Utrecht, The Netherlands; 2 Physical Therapy Research, department of Rehabilitation, Physical Therapy Science and Sport, Brain Center Rudolf Magnus, University Medical Center Utrecht, Utrecht, The Netherlands; 3 Department of Health Innovation and Technology, Fontys University of Applied Sciences, Eindhoven, The Netherlands; 4 Expertise Center Innovation of Care, Research Group Innovation of Mobility Care, University of Applied Sciences Utrecht, Utrecht, the Netherlands; 5 Department of General Practice, Julius Center for Health Sciences and Primary Care, University Medical Center Utrecht, Utrecht, The Netherlands

**Keywords:** Medically unexplained physical symptoms, Primary care, Screening method

## Abstract

**Background:**

Patients with medically unexplained physical symptoms (MUPS) are common in primary care, with a spectrum from mild to moderate and chronic MUPS. The burden of chronic MUPS is high, and early identification of moderate MUPS patients is important to prevent chronicity. The PRESUME screening method to identify moderate MUPS patients in primary care was developed, but insight in prognostic accuracy is needed. Therefore, our objective is to determine the prognostic accuracy for identification of moderate MUPS patients using the screening method with 5 year follow-up.

**Methods:**

The PRESUME screening method consists of three subsequent steps based on consultation frequency, exclusion of medical/psychiatric diagnosis and identification of MUPS. In a random 10% sample of patients from the Julius General Practitioners Network (*n* = 114.185), patients were identified with mild, moderate or chronic MUPS in 2008 (index year), using routine care data. In 5 years follow-up we calculated predictive values and odds ratio’s for sustained MUPS related symptoms.

**Results:**

In 2008, 789 patients (6.9% of the patient population) were identified as having mild, moderate or chronic MUPS. On average 55.5% of the moderate MUPS patients in 2008, still had MUPS related symptoms or developed chronic MUPS in 5 year follow-up. Positive predictive values for maintaining MUPS related symptoms or worsening was 67% after 1 year, and 48.7% after 5 years for moderate MUPS patients.

**Conclusion:**

The prognostic accuracy of the PRESUME screening method using electronic medical record data for identification of moderate MUPS patients is moderate. However, it might be a useful method to identify patients at increased risk of moderate MUPS, if combined with a validity check by the GP.

**Electronic supplementary material:**

The online version of this article (10.1186/s12875-019-0950-7) contains supplementary material, which is available to authorized users.

## Background

Medically unexplained physical symptoms (MUPS) are a serious problem in primary care [1]. Common unexplained symptoms in primary care include fatigue, pain, dizziness and general “malaise” [2]. In the Dutch multidisciplinary guideline for MUPS and Somatoform Disorders, MUPS are defined as physical complaints that last for at least a few weeks and are not explained by a medical condition after proper medical examination [3]. Of all complaints that patients present to their general practitioner (GP), 25–50% cannot be medically explained immediately [4].

MUPS can be regarded as a continuum with a spectrum from mild, to moderate, and persisting or chronic MUPS [3, 5, 6]. Seventy percent of the patients who consult their GP with a MUPS related diagnosis improve within 2 weeks (mild MUPS) [7–9]. The remaining 30% of the patients still experience unexplained symptoms after 3 months [9]. Most of them have moderate MUPS, the prevalence rate of patients with chronic MUPS (e.g. fibromyalgia, chronic fatigue syndrome or irritable bowel syndrome) in primary care is approximately 2.5% [4, 10].

Despite the low prevalence of chronic MUPS, the burden is substantial [4]. The impact on patients quality of life and daily functioning is high. Patients with MUPS have an above average consultation rate [11], and are more subject to diagnostic procedures [8]. For GPs adequate management of MUPS is challenging and often frustrating, due to the mismatch with the expectations of patients [12]. Finally, MUPS are associated with increased direct health care costs (due to higher utilization and unnecessary treatments) and indirect costs (e.g. work and insurance related costs) [11, 13].

Although previous research has identified several modifiable risk factors for the development of chronic MUPS [12, 14], GPs do not timely recognize patients with chronic MUPS [15]. It takes about 2 years before a chronic MUPS syndrome as fibromyalgia is diagnosed, without additional health benefits in the meantime [16]. Therefore, early identification of patients with increased risk of moderate MUPS is important to improve the prognosis, prevent chronicity and reduce health care costs. A screening method aiming at timely recognition of patients at increased risk of MUPS is needed. This could support so called ‘panel management’ [17] of MUPS in general practice, in which GPs identify patients with early stage MUPS and offer them interventions to prevent chronicity.

**Fig. 1:**
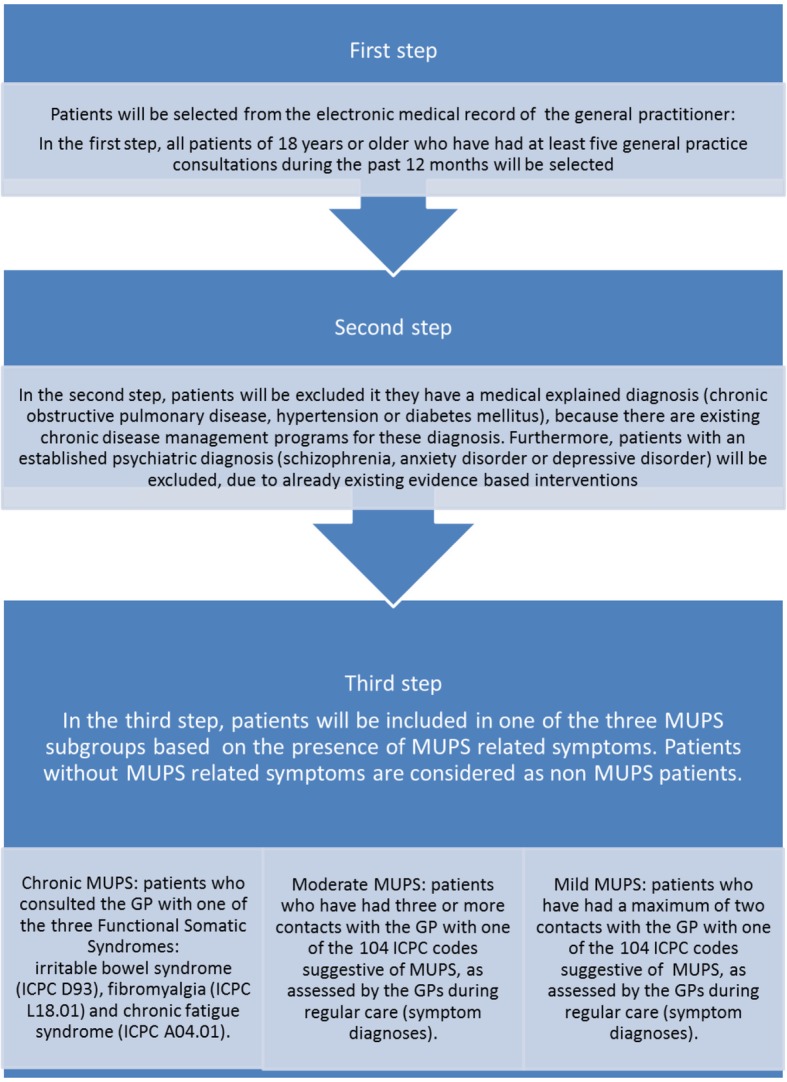
PRESUME screening method

Recently, a new screening method (PRESUME: preventive screening of medically unexplained physical symptoms) was developed to identify patients with an increased risk of mild, moderate or chronic MUPS using electronic medical record (EMR) data (Fig. 1). In a validation study in primary care, the screening method was compared with a questionnaire on the severity of somatic symptoms, demonstrating low sensitivity and high specificity [18]. However, this study focused only on the presence or absence of chronic MUPS. The prognostic accuracy of the PRESUME screening method for identification of patients with an increased risk of moderate MUPS remains unclear. Knowledge of the prognosis of patients with moderate MUPS is needed before the PRESUME screening method can be used for early identification of high risk patients and adequate prevention of chronicity. Furthermore, it is of interest to determine the consistency of the early identification of high risk patients by following the transition of patients between MUPS subgroups over time. Besides the transition of patients between MUPS subgroups over time, a part of the patients with MUPS will probably develop a medical or psychiatric diagnosis over time. Therefore, it is of interest to provide insight in the development of these disorders in patients of the MUPS subgroups.

Therefore, the objective of this study is to determine the prognostic accuracy of the PRESUME screening method in identifying patients with an increased risk of moderate MUPS. Secondly, transitions between MUPS subgroups for patients with an increased risk of moderate MUPS as well as transitions of MUPS subgroups to an anxiety and/or a depressive disorder or medical diagnosis over a 5 year follow-up period will be assessed.

## Method

### Study design

In this prognostic cohort study we identified patients with an increased risk of MUPS (mild, moderate and chronic) using historical data from electronic medical records of general practitioners, and followed them up over a period of 5 years to gain a prospective value of patients with moderate MUPS using PRESUME.

### Setting and study population

This study was conducted with routine health care data as collected within the Julius General Practitioners Network (JGPN) database, which was approved by the medical ethical committee of University Medical Center Utrecht (file#99–240). JGPN comprises data from 72 primary care practices with 215 GPs in the central part of the Netherlands. This represent the average Dutch primary care practice and GP, where 49% of the GPs is male with an average age of 48 years [19, 20]. Data in the JGPN database are anonymously extracted from the EMR from participating practices, and were successfully used in different studies [21–25], which is in line with the International Ethical Guidelines for Health-related Research Involving Humans and the Dutch Law on Medical Treatment Agreement [26]. Patients who deny access to their anonymized files when joining the practice are exempted from analysis (opt out). Other patients have given consent for using their anonymized data for scientific analysis.

GPs did not receive specific training on coding, but before EMR data was extracted, all included primary care practices signed a collaboration agreement that care registration is based on the standards and guidelines that apply within the profession of the GP [27]. GPs are systematically registering a clinical diagnosis using the Internal Classification of Primary Care (ICPC). Furthermore, the consultations are registered according to the “SOAP system” [28].

In 2008 the database consisted of 114.185 patients between 18 and 65 years. The patient population is a representative sample of the Dutch population [29]. Four times a year, the database is updated, adding new data to the previously retrieved data. Data was obtained from the data manager of the JGPN. The data manager conducted a data check, where a prerequisite for this study was that patients who had complete follow-up data during 5 year follow-up period (2009–2013) were eligible. To get a feasible database without unduly great statistical power, a random sample of 10% of the JGPN database in 2008 was used [30].

### Patients identification

The PRESUME screening method was used to identify patients with MUPS symptoms in three subgroups according to severity and disease impact. The method is based upon three subsequent steps (Fig. 1). In the first step patients aged ≥18 with five or more GP consultations in 2008 (the index year) were selected, since high consultation rate is a key phenomenon of MUPS in general practice [4]. In the second step patients with an established medical diagnosis, who were in a chronic disease management program for chronic obstructive pulmonary disease, hypertension or diabetes mellitus were excluded. Furthermore, patients with a psychiatric diagnosis were excluded due to already existing multidisciplinary guidelines with evidence based interventions for anxiety disorders, depressive disorders and schizophrenia [31–33]. In the third step, patients were identified with an increased risk of mild or moderate MUPS, based on the presence of MUPS related symptoms (Additional file 1), or chronic MUPS, based on an established chronic MUPS diagnosis (e.g. fibromyalgia, chronic fatigue syndrome or irritable bowel syndrome). All other patients were considered as non MUPS patients.

### Outcome

In order to assess the prognostic value for identifying an increased risk of sustained moderate MUPS, the index cohort (2008) were followed up for 5 years and reclassified according to the PRESUME in each follow-up year (2009–2013). Furthermore, the percentage of patients that developed a depressive and/or an anxiety disorder or a medical explained diagnosis (Additional file 2) during the 5 year follow-up period was determined.

### Data analysis

Data were analyzed using SPSS 22.0 for Windows (IBM Corporation, Armonk, NY, USA). Descriptive statistics were used to describe the patient population. Differences in baseline characteristics (gender, age) between subgroups were investigated using Pearson’s Chi-square and Kruskal-Wallis statistics.

For determination of the stability of the patients with an increased risk of moderate MUPS identified in the index year using PRESUME, transitions between MUPS subgroups over 5 years follow-up were determined, per year separately. It was hypothesized that at least 25% of the patients will still have an increased risk of mild or moderate MUPS (MUPS related symptoms) or developed chronic MUPS after 5 years follow-up.

To determine the prognostic value of PRESUME in predicting an increased risk of sustained MUPS diagnosis, positive and negative predictive values and odds ratios were calculated after one and 5 years follow-up. Accuracy was considered high when predictive values were > 75%, moderate accuracy with predictive values between 50 and 75% and low accuracy with predictive values < 50%.

Based on previous research [6, 34], our expectation was that at least 25% of the patients with chronic MUPS and 20% of the patients with an increased risk of moderate MUPS would be diagnosed with a depressive and/or an anxiety disorder during the 5 years follow-up. The patient was classified with a medical diagnosis if a medical diagnosis was coded during follow-up in the same ICPC chapter as the MUPS related diagnosis in the index year (Additional file 2). Based on previous research [35, 36], it was expected that less than 5% of the patients within one the MUPS subgroups will develop a medical diagnosis in the same ICPC chapter as the MUPS related diagnosis in the index year during the 5 years follow-up. Differences were investigated using one-way ANOVA statistics. To determine the prognostic risk for a depressive and/or an anxiety disorder or medical diagnosis odds ratios were calculated.

## Results

Of the random sample of 11.419 patients from the JGPN database (50.6% female, mean age 41.7 years), 2.073 patients (18.2%) had more than five encounters in 2008. Of these, 35.1% (*n* = 729) had a medical explained diagnosis (e.g. chronic obstructive pulmonary disease, hypertension or diabetes mellitus) or an established psychiatric diagnosis (e.g. schizophrenia, anxiety disorder or depressive disorder). Of the remaining 1344 patients, 789 (58.7%) were identified with an increased risk of MUPS and classified in one of the MUPS subgroups (see Table 1). Of the total sample, 455 patients (4%) were identified in the mild MUPS group (69.9% female, mean age 41.4 years), 273 patients (2.4%) were identified with an increased risk of moderate MUPS (70% were female, mean age 41.1 years) and 61 patients (0.5%) were identified in the chronic MUPS group (73.8% female, mean age 42.5 years).

**Table 1:** Baseline characteristics of the study population in index year

	Study population	Chronic MUPS	Moderate MUPS	Mild MUPS	Non MUPS	Significance
	(*n* = 11.419)	(*n* = 61; 0.5%)	(*n* = 273; 2.4%)	(*n* = 455; 4.0%)	(n = 10.630; 93.1%)	(*p*-value)
Female, n (%)	5.779 (50.6%)	45 (73.8%)	191 (70%)	318 (69.9%)	5.225 (49.2%)	< 0.001^a^
Mean age in years (SD)	41.7 (12.5)	42.5 (11.9)	41.1 (12.0)	41.4 (11.9)	41.8 (12.5)	> 0.05^b^

^a^Differences between MUPS classifications evaluated with Pearson’s Chi-square test.^b^Differences between MUPS classifications evaluated with Kruskal-Wallis test

Of the patients identified with an increased risk of moderate MUPS in 2008, 46% still had MUPS related symptoms during the 5 year follow-up period, and 9.5% (*n* = 26) had developed chronic MUPS (see Table 2).

**Table 2:** Percentages of changes of moderate MUPS patients (*n* = 273) in index year (2008) during 5 years follow-up

	2009% (n)	2010% (n)	2011% (n)	2012% (n)	2013% (n)
	One year follow-up	Two years follow-up	Three years follow-up	Four years follow-up	Five years follow-up
Non MUPS	33 (90)	38.5 (105)	46.9 (128)	52.4 (143)	51.3 (140)
Mild MUPS	31.9 (87)	31.1 (85)	27.8 (76)	19.4 (53)	21.6 (59)
Moderate MUPS	34.1 (93)	26.4 (72)	18.3 (50)	19.8 (54)	17.6 (48)
Chronic MUPS	1.1 (3)	4.0 (11)	7.0 (19)	8.4 (23)	9.5 (26)

The prognostic value of patients identified at increased risk of moderate MUPS in 2008 was determined after 1 year and after 5 years follow-up (see Table 3). The positive predictive value (PPV) for still having MUPS after 1 year follow-up was 67%. The negative predictive value (NPV) was 82.5% after 1 year. After 5 years, the PPV was 48.7% and the NPV was 77.8%. Patients identified at increased risk of moderate MUPS have 9.8 higher odds of maintaining MUPS related symptoms or worsening in 1 year follow-up compared to patients with non MUPS. After 5 years follow-up, the odds for sustained MUPS related symptoms or progression to chronic MUPS is 3.3 times higher for patients identified at increased risk of moderate MUPS compared to patients with non MUPS in the index year.

**Table 3:** Prognostic accuracy for moderate MUPS patients after one and 5 years follow-up

	non MUPS / maintained or deteriorated (2009)			non MUPS / maintained or deteriorated (2013)		
	Positive Predictive value	Negative Predictive value	Odds ratio	Positive Predictive value	Negative Predictive value	Odds ratio
	(95% CI)	(95% CI)	(95% CI)	(95% CI)	(95% CI)	(95% CI)
Moderate MUPS in 2008; *n* = 273	0.670 (0.614–0.726)	0.825 (0.821–0.835)	9.82 (7.59–12.70)	0.487 (0.427–0.546)	0.778 (0.770–0.786)	3.33 (2.62–4.24)

During the follow-up period, 261 patients of the index sample (2.2%) developed a depressive and/or an anxiety disorder, of which a depressive disorder was most frequently diagnosed (*n* = 145; 55.5%) (see Table 4). Additionally, 109 patients developed both an anxiety disorder and a depressive disorder. Of all patients identified at increased risk of moderate MUPS in 2008 (*n* = 273), 13.5% (*n* = 37) developed a depressive and/or an anxiety disorder in 5 years follow-up, compared to 1.4% (*n* = 156) of the patients without MUPS, 12.3% (*n* = 56) of the patients identified at increased risk of mild MUPS and 19.6% (*n* = 12) of the patients with chronic MUPS (see Table 4).

**Table 4:** Depressive and/or anxiety disorder and medical diagnosis for patients in subgroups during 5 years follow-up

	Depressive and/or an anxiety disorder during follow-up; *n* = 261						Medical explained diagnoses during follow-up; *n* = 337	
	Anxiety disorder		Depressive disorder		Anxiety and depressive disorder			
	% (*n*)	OR (95% CI)	% (*n*)	OR (95% CI)	% (*n*)	OR (95% CI)	% (*n*)	OR (95% CI)
Chronic MUPS in 2008; n = 61	9.8 (6)	18.67 (7.72–45.15)^a^	8.1 (5)	11.01 (4.28–28.29)^a^	1.6 (1)	49.01 (5.38–446.41)	16.4 (10)	8.82 (4.42–17.60)^a^
Moderate MUPS in 2008; n = 273	6.2 (17)	10.98 (6.33–19.05)^a^	6.9 (19)	8.68 (5.20–14.49) ^a^	0.4 (1)	10.17 (1.13–91.38)	15.8 (43)	8.41 (5.92–11.95)^a^
Mild MUPS in 2008; *n* = 455	4.4 (20)	7.64 (4.57–12.76)^a^	7.0 (32)	8.65 (5.70–13.12) ^a^	0.9 (4)	24.07 (5.99–96.61)	11.6 (53)	5.93 (4.33–8.13)^a^
Non MUPS in 2008; *n* = 10.630	0.6 (63)	–	0.9 (89)	–	0.0 (4)		2.1 (231)	–

^a^ There is a significant difference between the MUPS subgroups and non MUPS group on the development of a depressive and/or an anxiety disorder or a medical explained diagnosis, *p* < 0.05

Of the 11.419 patients, 337 patients (2.9%) were diagnosed with a confirmed medically diagnosis during follow-up (see Table 4). Of the patients within the moderate MUPS subgroup in 2008 (*n* = 273), 15.8% (*n* = 43) developed a medical explained diagnosis in the same ICPC chapter as the MUPS related symptoms in the index year, as compared to 2.1% (*n* = 231) of the patients without MUPS, 11.6% (*n* = 53) of the patients identified at increased risk of mild MUPS and 16.4% (*n* = 10) of the patients with chronic MUPS during 5 years follow-up. Of all patients who developed a medical diagnosis during follow-up, most diagnosis regarded in the ICPC chapter L (musculoskeletal). The risk for development of a medical diagnosis in patients within one of the MUPS subgroups is significantly higher compared with the non MUPS group.

## Discussion

The PRESUME screening demonstrated moderate prognostic accuracy for sustained MUPS related symptoms after 1 year and low to moderate accuracy after 5 years. Over a period of 5 years, more than 50% of the patients identified at increased risk of moderate MUPS had sustained MUPS related symptoms. Our findings indicate that the prognostic value of the PRESUME screening method is representative in patients with moderate MUPS without restrictions for the duration of complaints. The PRESUME method could support MUPS panel management in primary care, by combining early identification of moderate MUPS patients followed by a targeted intervention program to prevent chronicity.

The included study population is a representative sample of the Dutch population [37]. In the MUPS subgroups there is an overrepresentation of females, which is in line with other studies [10, 38]. Furthermore, the population in the JGPN database is also comparable to the Dutch population regarding urbanization and age [21].

Almost 20% of the patients with chronic MUPS and almost 15% of those identified at increased risk of moderate MUPS developed a depressive and/or an anxiety disorder in 5 year follow-up, which confirms a higher risk for mood disorders in patients with MUPS, as reported earlier [34, 39–41]. The percentage was lower compared to other studies [6, 34], which may be explained by the fact that patients with an existing diagnosis of a depressive and/or an anxiety disorder were excluded in step 2 of the PRESUME screening method. A disadvantage of excluding patients with a depressive and/or an anxiety disorder is that we also excluded MUPS patients with a mood disorder. However, according to the Dutch multidisciplinary guidelines this MUPS subgroup has specific treatment recommendations, which legitimates the exclusion in the PRESUME screening method [31, 32].

Of all patients within one of the MUPS subgroups in 2008, we hypothesized that less than 5% would develop a medical diagnosis during 5 years. Our results proved otherwise: 11.6% (*n* = 53) of the patients identified at increased risk of mild MUPS in 2008, 15.8% (*n* = 43) of those identified at increased risk of moderate MUPS and 16.4% (*n* = 10) of the patients with chronic MUPS was labelled with a medical diagnosis in the same ICPC chapter as in which they had MUPS in 2008, during the 5 years follow-up. In short, almost 50% of the patients identified at increased risk of MUPS will develop a medical diagnosis. However, this percentage is probably an overestimation since 54 of the 106 patients (50.9%) who were diagnosed with a medical diagnosis during follow-up, also still had MUPS related symptoms. Consequently, the MUPS related symptoms cannot be explained by the medical diagnosis, and the medical diagnosis seems not always anatomically be related to the MUPS related symptoms. Nevertheless, patients identified at increased risk of moderate MUPS according to the PRESUME screening method might have an established medical diagnosis, since we only exclude patients with chronic obstructive pulmonary disease, hypertension or diabetes mellitus in the second step of the PRESUME screening method. Therefore, to ensure that we have identified patients in the right stage of MUPS, also due to the moderate prognostic accuracy, GPs should perform a validity check and filter out patients with an established medical diagnosis, to prevent that patients are incorrectly offered treatment for MUPS.

Our study has some strengths and limitations. A first strength is that we were able to analyze data from a large primary care cohort with routine care data, which makes our findings generalizable to other general practices in the Netherlands. Another strength is that this is the first study, as far as we know, that has focused on identifying and follow-up of patients identified at increased risk of moderate MUPS using electronic medical record data. Most studies so far focus on patients with chronic MUPS, while those identified at increased risk of moderate MUPS may be a better target for preventive interventions [18, 42, 43]. Besides the strengths, we also should note some limitations.

First, the PRESUME screening method is over inclusive since it is developed to identify patients at increased risk of having MUPS in the primary care patient population. Therefore, the selected ICPC codes of step 3 of the PRESUME screening method are diagnoses which have a higher risk of staying unexplained. As a consequence, the selection does lead to false positive and negative patients, and an additional check by the GP might be useful before inviting selected patients for a preventive intervention program. Second, there may be a possible underestimation of the number of patients that has developed chronic MUPS, since GPs are reluctant to diagnose a chronic MUPS syndrome in their strive to prevent further somatisation [44]. The low prevalence of chronic MUPS might also partly explain the low to moderate positive predictive value of our screening method in long term follow-up [45]. A third limitation is the possible variation in the data, since the data have been extracted from electronic files of participating practices and therefore depends on quality of GP registration. In the Netherlands, GPs have a specific guideline on adequate care registration with diagnosis patients using the ICPC as well as registration according to the “SOAP system” [27, 28]. Despite this guideline, the data may still be sensitive for registration errors. Therefore, our advice is to conduct a validity check by the GP, after patients are identified at increased risk of moderate MUPS according to the PRESUME screening method. A fourth limitation is the possibility of selection bias due to the eligibility criterion of having complete follow-up data, as well as the search for explanatory medical diagnosis in the same ICPC chapter as the MUPS related diagnosis in 2008. In this way, we did miss patients who moved and switched GP during follow-up, and we may have missed diagnoses in other ICPC chapter that explained the original MUPS symptomatology. Furthermore, patients who were diagnosed with a depressive and/or an anxiety disorder, according to step two of the PRESUME screening method, were excluded and classified as non MUPS patients. This might also be a potential form of selection bias due to the known association between MUPS and an anxiety and/or depressive disorder [6, 34].

The prognostic accuracy for patients identified at increased risk of moderate MUPS according to the PRESUME screening method is moderate in early identification of patients with increased risk of moderate MUPS in primary care. An average of more than 50% of the patients who were identified with increased risk of moderate MUPS in 2008 are still consulting the GP at least five times a year with at least one MUPS related symptom during 5 years follow-up. This means that in a large proportion of patients identified with increased risk of moderate MUPS, the burden stays high with high consultation rate and impact on patients quality of life, as well as for GPs with challenging consultations, difficulties in identifying patients with MUPS, and doubts to pursue further diagnostic evaluation, leading to a deteriorating doctor-patient relationship [11, 12]. GPs found adequate management of MUPS challenging and they mainly focus on maintaining the doctor-patient relationship when patients keep presenting with MUPS [46]. Therefore, for both patients and GPs in primary care it is of interest to identify patients with increased risk of moderate MUPS. The PRESUME screening method can support timely pattern recognition by the GP. After the identification of patients with moderate MUPS according to the PRESUME screening method, the GP can conduct a validity check and patients with an established medical diagnosis can be excluded as having an increased risk of moderate MUPS. Furthermore, the GP can exclude patients in which further diagnostic evaluation of the symptoms is needed. The identification of patients with moderate MUPS can support adequate management of patients with MUPS as well as the doctor-patient relation, since GPs can conduct a more comprehensive bio-psychosocial approach in their consultations [47]. In addition, the identification of patients with MUPS can support a more proactive a panel management approach. Patients at risk can be actively approached by their GP, offering them a preventive intervention program. The intervention should focus on improving illness perception and self-management, contribute to a better recovery of the moderate MUPS symptoms and prevent chronic MUPS. Future research should focus on the development of this intervention and asses its effectiveness.

## Conclusion

The prognostic accuracy of the PRESUME screening method using electronic medical record data for identification of moderate MUPS patients is moderate. However, it might be a useful method to identify patients at increased risk of moderate MUPS, if combined with a validity check by the GP.

## Additional files

Additional file 1: 104 ICPC codes refer to MUPS related diagnoses. A list of 104 International Classification of Primary Care codes of MUPS related symptoms used in the third step in the PRESUME screening method to identify patients with mild or moderate MUPS. (DOCX 20 kb)

Additional file 2: Medically explained diagnoses. A list of International Classification of Primary Care codes of medically explained diagnoses used to determine the percentage of patients that developed a medically explained diagnosis during the 5 year follow-up period. (DOCX 20 kb)

## Abbreviations

EMR: Electronic medical record
GP: General Practitioner
ICPC: International classification of primary care code
JGPN: Julius General Practitioners Network
MUPS: Medically Unexplained Physical Symptoms
NPV: Negative predictive value
PPV: Positive predictive value
PRESUME: Preventive screening of medically unexplained physical symptoms
