# Supplementary material for: Identification of patients with moderate medically unexplained physical symptoms in primary care with a five years follow-up
Source: BMC Fam Pract. 2019 May 21;20:66. doi: 10.1186/s12875-019-0950-7 (PMC6530058; doi:10.1186/s12875-019-0950-7)
Supplement: Supplementary file 1 — 104 ICPC codes refer to MUPS related diagnoses. A list of 104 International Classification of Primary Care codes of MUPS related symptoms used in the third step in the PRESUME screening method to identify patients with mild or moderate MUPS. (DOCX 20 kb) [file 12875_2019_950_MOESM1_ESM.docx]

**Additional file 1** 104 ICPC codes refer to MUPS related diagnoses

**Abdomen**D01 Abdominal pain/ cramps general
D02 Abdominal pain epigastric
D04 Rectal/ anal pain
D06 Abdominal pain localized other
D08 Flatulence/ gas/ belching
D09 Nausea
D11 Diarrhoea
D12 Constipation
D18 Change faeces/ bowel movements
D93 Irritable bowel syndrome
T03 Loss of appetite
T08 Weight loss

**Fatigue**A04 Weakness/tiredness general
 .01 Chronic fatigue syndrome

**Musculoskeletal**L01 Neck symptom/ complaint
L02 Back symptom/ complaint
L03 Low back symptom/ complaint
L05 Flank symptom/ complaint
L06 Axilla symptom/ complaint
L07 Jaw symptom/ complaint
L08 Shoulder symptom/ complaint
L09 Arm symptom/ complaint
L10 Elbow symptom/ complaint
L11 Wrist symptom/ complaint
L12 Hand/ finger symptom/ complaint
L13 Hip symptom/ complaint
L14 Leg/ thigh symptom/ complaint
L15 Knee symptom/ complaint
L16 Ankle symptom/ complaint
L17 Foot/ toe symptom/ complaint
L18 Muscle pain
 .01 Fibromyalgia
L79 Sprain/ strain of joint NOS
 .01 Whiplash trauma cervical spine

**Cardiology-Respitaroy**K01 Heart pain
K02 Pressure/ tightness of heart
K03 Cardiovasculair pain NOS
K04 Palpitations/ awareness of heart
K05 Irregular heartbeat other
L04 Chest symptom/ complaint

**(Pseudo-)Neurology and ENT**A01 Pain gereral/ multiple sites
F13 Eye sensation abnormal
H02 Hearing complaint
H03 Tinnitus, ringing/buzzing ear
N01 Headache
N02 Tension headache
N03 Pain face
N05 Tingling fingers/feet/toes
N17 Vertigo/dizziness
 .01 Sensation of unsteadiness
 .02 Lightheadedness

**Other**S01 Pruritis
R98 Hyperventilation syndrome

Psychiatry
A26 Fear of cancer NOS
A27 Fear of other disease NOS
B25 Fear of aids/ HIV
B26 Fear cancer blood/ lymph
B27 Fear blood/ lymph disease other
D26 Fear of cancer of digestive system
D27 Fear of digestive disease other
F27 Fear of eye disease
H27 Fear of ear disease
K24 Fear of heart disease
K25 Fear of hypertension
K27 Fear cardiovascular disease other
L26 Fear of cancer musculoskeletal
L27 Fear musculoskeletal disease other
N26 Fear cancer neurological system
N27 Fear of neurological disease other
P01 Feeling anxious/nervous/tense
P06 Sleep disturbance
P75 Somatization disorder
R26 Fear of cancer respiratory system
R27 Fear of respiratory disease other
S26 Fear of cancer of skin
S27 Fear of skin disease other
T26 Fear of cancer of endocrine system
T27 Fear endocrine/metabolic dis other
U26 Fear of cancer of urinary system
U27 Fear of urinary disease other
X23 Fear sexually transmitted disease (f)
X24 Fear of sexual dysfunction female
X25 Fear of genital cancer female
X26 Fear of breast cancer female
Y24 Fear of sexual dysfunction male
Y25 Fear sexually transmitted dis. Male
Y26 Fear of genital cancer male
Y27 Fear of genital disease male other
Z29.01 Burnout / stress

Urological/ Genital complaints
U02 Urinary frequency/urgency
U05 Urination problems other
X01 Genital pain female
X02 Menstrual pain
X03 Intermenstrual pain
X04 Painful intercourse female
X09 Premenstrual symptom/complaint
X11 Menopausal symptom/complaint
X15 Vaginal symptom/complaint other
X16 Vulval symptom/complaint
X17 Pelvis symptom/complaint female
Y01 Pain in penis
Y02 Pain in testis/scrotum
Y04 Penis symptom/complaint other
Y08 Sexual function symptom/ complaint (m)

**Abdomen**D01 Abdominal pain/ cramps general
D02 Abdominal pain epigastric
D04 Rectal/ anal pain
D06 Abdominal pain localized other
D08 Flatulence/ gas/ belching
D09 Nausea
D11 Diarrhoea
D12 Constipation
D18 Change faeces/ bowel movements
D93 Irritable bowel syndrome
T03 Loss of appetite
T08 Weight loss

**Fatigue**A04 Weakness/tiredness general
 .01 Chronic fatigue syndrome

**Musculoskeletal**L01 Neck symptom/ complaint
L02 Back symptom/ complaint
L03 Low back symptom/ complaint
L05 Flank symptom/ complaint
L06 Axilla symptom/ complaint
L07 Jaw symptom/ complaint
L08 Shoulder symptom/ complaint
L09 Arm symptom/ complaint
L10 Elbow symptom/ complaint
L11 Wrist symptom/ complaint
L12 Hand/ finger symptom/ complaint
L13 Hip symptom/ complaint
L14 Leg/ thigh symptom/ complaint
L15 Knee symptom/ complaint
L16 Ankle symptom/ complaint
L17 Foot/ toe symptom/ complaint
L18 Muscle pain
 .01 Fibromyalgia
L79 Sprain/ strain of joint NOS
 .01 Whiplash trauma cervical spine

**Cardiology-Respitaroy**K01 Heart pain
K02 Pressure/ tightness of heart
K03 Cardiovasculair pain NOS
K04 Palpitations/ awareness of heart
K05 Irregular heartbeat other
L04 Chest symptom/ complaint

**(Pseudo-)Neurology and ENT**A01 Pain gereral/ multiple sites
F13 Eye sensation abnormal
H02 Hearing complaint
H03 Tinnitus, ringing/buzzing ear
N01 Headache
N02 Tension headache
N03 Pain face
N05 Tingling fingers/feet/toes
N17 Vertigo/dizziness
 .01 Sensation of unsteadiness
 .02 Lightheadedness

**Other**S01 Pruritis
R98 Hyperventilation syndrome

Psychiatry
A26 Fear of cancer NOS
A27 Fear of other disease NOS
B25 Fear of aids/ HIV
B26 Fear cancer blood/ lymph
B27 Fear blood/ lymph disease other
D26 Fear of cancer of digestive system
D27 Fear of digestive disease other
F27 Fear of eye disease
H27 Fear of ear disease
K24 Fear of heart disease
K25 Fear of hypertension
K27 Fear cardiovascular disease other
L26 Fear of cancer musculoskeletal
L27 Fear musculoskeletal disease other
N26 Fear cancer neurological system
N27 Fear of neurological disease other
P01 Feeling anxious/nervous/tense
P06 Sleep disturbance
P75 Somatization disorder
R26 Fear of cancer respiratory system
R27 Fear of respiratory disease other
S26 Fear of cancer of skin
S27 Fear of skin disease other
T26 Fear of cancer of endocrine system
T27 Fear endocrine/metabolic dis other
U26 Fear of cancer of urinary system
U27 Fear of urinary disease other
X23 Fear sexually transmitted disease (f)
X24 Fear of sexual dysfunction female
X25 Fear of genital cancer female
X26 Fear of breast cancer female
Y24 Fear of sexual dysfunction male
Y25 Fear sexually transmitted dis. Male
Y26 Fear of genital cancer male
Y27 Fear of genital disease male other
Z29.01 Burnout / stress

Urological/ Genital complaints
U02 Urinary frequency/urgency
U05 Urination problems other
X01 Genital pain female
X02 Menstrual pain
X03 Intermenstrual pain
X04 Painful intercourse female
X09 Premenstrual symptom/complaint
X11 Menopausal symptom/complaint
X15 Vaginal symptom/complaint other
X16 Vulval symptom/complaint
X17 Pelvis symptom/complaint female
Y01 Pain in penis
Y02 Pain in testis/scrotum
Y04 Penis symptom/complaint other
Y08 Sexual function symptom/ complaint (m)
