# Supplementary material for: Identification of patients with moderate medically unexplained physical symptoms in primary care with a five years follow-up
Source: BMC Fam Pract. 2019 May 21;20:66. doi: 10.1186/s12875-019-0950-7 (PMC6530058; doi:10.1186/s12875-019-0950-7)
Supplement: Supplementary file 2 — Medically explained diagnoses. A list of International Classification of Primary Care codes of medically explained diagnoses used to determine the percentage of patients that developed a medically explained diagnosis during the 5 year follow-up period. (DOCX 20 kb) [file 12875_2019_950_MOESM2_ESM.docx]

Additional file 2: Medically explained diagnoses

**Chapter A**A70 Tuberculosis
A75 Infectious mononucleosis
A77 Viral disease other/NOS
A78.05 Lyme disease, Lyme borreliosis
A79 Malignancy NOS
A86 Toxic effect non-medicinal substance
A91.06 subclinical hypothyroidism
A91.07 subclinical hyperthyroidism
 **Chapter B**B72 Hodgkin's disease/lymphoma
B72.01 Hodgkin's disease
B72.02 Non-Hodgkin lymphoma
B73 Leukemia
B74 Malignant neoplasm blood other
B74.01 Multiple myeloma
B77 Injury blood/lymph/spleen other
B78 Hereditary haemolytic anaemia
B78.01 thalassaemia
B78.02 sickle-cell anaemia
B78.03 Anemia G6PD deficiency
B79 Congenital anomaly blood/lymph other
B80 Iron deficiency anaemia
B81 Anaemia, Vitamin B12/folate def
B81.01 folate deficiency
B81.02 Anaemia vit B12
B82 Anaemia other/unspecified
B90 HIV-infection/AIDS
B90.02 AIDS/ARC
 **Chapter D**D72.03 Acute hepatitis C
D72.05 Carrier hepatitis C / chronic hepatitis C
D74 Malignant neoplasm stomach
D75 Malignant neoplasm colon/rectum
D76 Malignant neoplasm pancreas
D77 Malig. neoplasm digest other/NOS
D77.01 Malignant esophagus
D77.02 Malignant salivary glands
D77.03 Malignant lip / mouth / tongue
D77.04 Malignant liver / gallbladder / biliary
D84.03 oesophagial reflux with oesophagitis
D86 Peptic ulcer other
D86.01 Ulcus ventriculi
D94 Chronic enteritis/ulcerative colitis
D94.01 Ulcerative colitis
D94.02 Crohn's disease
D99.06 Celiac Disease

**Chapter F**F74 Neoplasm of eye/adnexa
F74.01 Malignant eye/adnexa

**Chapter H**H75 Neoplasm of ear
H75.01 Malignant ear
 **Chapter K**K72 Neoplasm cardiovascular
K72.01 Malignant cardiovascular
K74 Ischaemic heart disease w. angina
K74.01 Unstable angina
K74.02 Stable angina
K75 Acute myocardial infarction
K77 Decompensatio cordis

K77.01 Acuut decompensatio cordis/astma cardiale

K77.02 Chronic decompensatio cordis

K78 Atrial fibrillation/flutter

K79 Paroxysmal tachycardia

K79.01 supraventricular tachycardia

K79.02 ventricular tachycardia

K80 Cardiac arrhythmia NOS

K80.01 supraventricular extrasystoles

K80.02 ventricular extrasystoles

K80.03 Sick sinus syndrome

**Chapter L**L70 Infection of musculoskeletal system
L70.01 osteomyelitis
L70.02 septic arthritis
L71 neoplasm musculoskeletal
L71.01 Malignant neoplasm musculoskeletal
L76.06 Fracture spine
L76.07 Fracture pelvis
L83.01 herniated cervical
L84 Osteoarthritis / spine spondylosis
L86.01 HNP (thoracic / lumbar)
L88 Rheumatoid/seropositive arthritis
L88.01 Rheumatoid arthritis
L88.02 Morbus Bechterew (ankylosing spondylitis)
L89 Osteoarthrosis of hip
L90 Osteoarthrosis of knee
L99.06 Tietze Syndrome
L99.12 Polymyalgia rheumatica

**Chapter N**N74 Malignant neoplasm nervous system
N75 Benign neoplasm nervous system
N76 Neoplasm nervous system unspec.
N86 Multiple sclerosis
N87 Parkinsonism
N87.01 Parkinson's disease
N91 Facial paralysis/bell's palsy
N92 Trigeminal neuralgia
N94.01 Guillain-Barré syndrome
N99.01 ALS
N99.02 Myasthenia gravis
N99.03 Muscular dystrophy

**Chapter P**Not applicable
 **Chapter R**R83.02 Sarcoïdosis
R84 Malignant neoplasm bronchus/lung
R85 Malignant neoplasm respiratory, other
R95 Chronic obstructive pulmonary disease
R96 Astma
R96.01 reactive airways disease

**Chapter S**S77 Malignant neoplasm of skin
S77.01 basal cell carcinoma
S77.02 squamous cell / squamous cell carcinoma
S77.03 Malignant melanoma
S77.04 Kaposi's sarcoma
 **Chapter T**T71 Malignant neoplasm thyroid
T72 Benign neoplasm thyroid
T73 Neoplasm endocrine oth/unspecified
T85 Hyperthyroidism/thyrotoxicosis
T86 Hypothyroidism/myxoedema
T90.01 juvenile onset diabetes; type 1 diabetes
T90.02 late onset diabetes; type 2 diabetes
T99.08 Cushing's syndrome
T99.09 Addison’s syndrome

**Chapter U**U75 Malignant neoplasm of kidney
U76 Malignant neoplasm of bladder
U77 Malignant neoplasm urinary other
U79 Neoplasm urinary tract NOS

**Chapter W**Not applicable

**Chapter X**X75 Malignant neoplasm cervix
X76 Malignant neoplasm breast female
X76.01 Adenocarcinoma mom female
X77 Malignant neoplasm genital other (f)
X77.01 Endometrial cancer
X77.02 Malignant ovary

**Chapter Y**Y77 Malignant neoplasm prostate
Y78 Malignant neoplasm male genital other
Y78.01 Malignant penis
Y78.02 Malignant testis
Y78.03 Malignant breast
